# Combined 6-benzylaminopurine and H_2_O_2_ stimulate the astaxanthin biosynthesis in *Xanthophyllomyces dendrorhous*

**DOI:** 10.1007/s00253-023-12875-9

**Published:** 2024-01-22

**Authors:** Alejandro Torres-Haro, Jorge Verdín, Manuel R. Kirchmayr, Melchor Arellano-Plaza

**Affiliations:** Biotecnología Industrial, Centro de Investigación y Asistencia en Tecnología y Diseño del Estado de Jalisco, A.C. Camino Arenero 1227, El Bajío del Arenal, 45019 Zapopan, Jalisco Mexico

**Keywords:** Carotenoids, Phytohormones, Oxidative stress, Stimulation, Cell viability, Transcriptional responses

## Abstract

**Abstract:**

Astaxanthin is one of the most attractive carotenoids due to its high antioxidant activity and beneficial biological properties, while *Xanthophyllomyces dendrorhous* is one of its main microbial sources. Since astaxanthin is synthesized as a response to oxidative stress, several oxidative agents have been evaluated to increase *X. dendrorhous* astaxanthin yields. However, the extent of the stimulation is determined by the cellular damage caused by the applied oxidative agent. Phytohormones have also been reported as stimulants of astaxanthin biosynthesis acting directly on its metabolic pathway and indirectly promoting cellular resistance to reactive oxygen species. We reasoned that both oxidative agents and phytohormones lead to increased astaxanthin synthesis, but the latter could mitigate the drawbacks of the former. Thus, here, the stimulation on astaxanthin biosynthesis, as well as the cellular and transcriptional responses of wild type *X. dendrorhous* to phytohormones (6-benzylaminopurine, 6-BAP; abscisic acid, ABA; and indole-3-acetic acid, IAA), and oxidative agents (glutamate, menadione, H_2_O_2,_ and/or Fe^2+^) were evaluated as a single or combined treatments. ABA and 6-BAP were the best individual stimulants leading to 2.24- and 2.60-fold astaxanthin biosynthesis increase, respectively. Nevertheless, the effect of combined 6-BAP and H_2_O_2_ led to a 3.69-fold astaxanthin synthesis increase (0.127 ± 0.018 mg astaxanthin/g biomass). Moreover, cell viability (> 82.75%) and mitochondrial activity (> 82.2%) remained almost intact in the combined treatment (6-BAP + H_2_O_2_) compared to control (< 52.17% cell viability; < 85.3% mitochondrial activity). On the other hand, mRNA levels of *hmgR*, *idi*, *crtYB*, *crtR*, and *crtS*, genes of the astaxanthin biosynthetic pathway, increased transiently along *X. dendrorhous* fermentation due to stimulations assayed in this study.

**Key points:**

• *Combined 6-BAP and H*_*2*_*O*_*2*_* is the best treatment to increase astaxanthin yields in X. dendrorhous.*

• *6-BAP preserves cell integrity under oxidative H*_*2*_*O*_*2*_* stress conditions.*

• *6-BAP and H*_*2*_*O*_*2*_* increase transcriptional responses of hmgR, idi, and crt family genes transiently.*

**Graphical abstract:**

**Figure:**
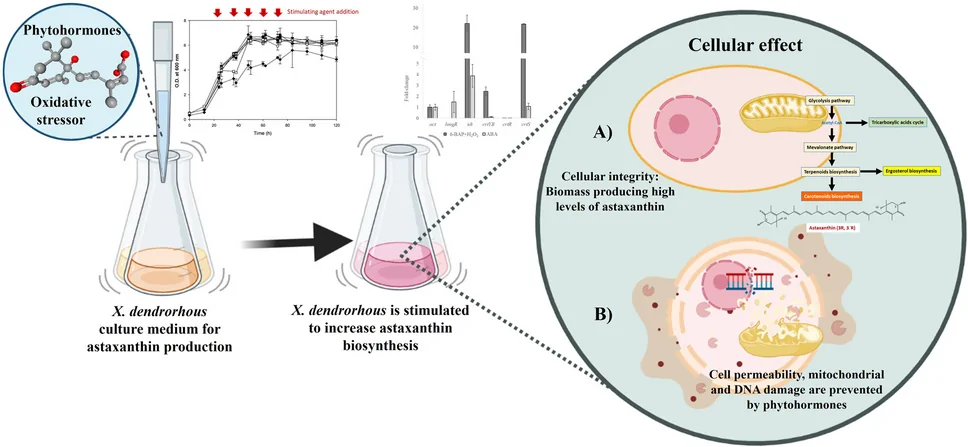

**Supplementary Information:**

The online version contains supplementary material available at 10.1007/s00253-023-12875-9.

## Introduction

Carotenoids are terpenoid pigments with powerful biological functions such as antioxidants, UV radiation protectants, and vitamin A precursors, capable of preventing degenerative diseases (cancer, hypertension, diabetes, etc.) (Arunkumar et al. 2020; Avalos and Limón 2015; Mata-Gómez et al. 2014; Miki 1991). These compounds are widespread in nature being responsible for the red–orange colorations of some fruits and flowers, but also salmon, flamingos, or crustaceans (Amado and Vázquez 2015; Avalos and Limón 2015; Domonkos et al. 2013; Mata-Gómez et al. 2014).

Due to their important biological properties, carotenoids such as phytoene, lycopene, β-carotene, zeaxanthin, and astaxanthin have an increasing demand in the food, pharmaceutical, and cosmetic industries (Bhosale 2004; Leu and Boussiba 2014; Mata-Gómez et al. 2014; Nutakor et al. 2022; Wang et al. 2015). Astaxanthin is one of the most attractive carotenoids due to its high antioxidant activity, 10 and 100 times higher than β-carotene and α-tocopherol, respectively (Miki 1991; Mortensen et al. 2001).

The astaxanthin price depends on its provenance (synthetic or natural) and purity. In 2007, chemically synthesized astaxanthin had an annual turnover of more than 200 million USD and a sales price of around $2500 USD/kg. Due to its enantiomeric composition, natural astaxanthin shows a greater biological activity compared to the synthetic one, thus the price of the former is 40-fold higher than the average (Østerlie et al. 2000; Storebakken et al. 1987; Torres-Haro et al. 2021a; Domínguez-Bocanegra et al. 2007; Higuera-Ciapara et al. 2006).

*Xanthophyllomyces dendrorhous* (*Phaffia rhodozyma*) and *Haematococcus pluvialis* are the main natural producers of astaxanthin (Domínguez-Bocanegra et al. 2007; Higuera-Ciapara et al. 2006; Leu and Boussiba 2014; Li et al. 2022; Mata-Gómez et al. 2014; Torres-Haro et al. 2021a; Zhang et al. 2020). *Xanthophyllomyces dendrorhous* is preferred for large-scale production since it synthesizes non-esterified astaxanthin as its main carotenoid (85% of total) at 20 °C and pH 6.0 (Kim et al. 2005; Liu and Wu 2007; Ramírez et al. 2001; Rodríguez-Sáiz et al. 2010; Torres-Haro et al. 2021b; Wang et al. 2019). However, the astaxanthin production levels by wild type *X. dendrorhous* are low (170–200 µg astaxanthin/g of dry biomass) and uncompetitive compared to chemical synthesis (Andrewes et al. 1976; Li et al. 2022; Schmidt et al. 2011; Torres-Haro et al. 2021b; Visser et al. 2003).

Several strategies to increase astaxanthin production by *X. dendrorhous* have been explored: (i) highly assimilable and low-cost culture media and (ii) bioprocess engineering, and iii) generation of mutant overproducing strains (Gassel et al. 2013; Martínez-Moya et al. 2022; Schmidt et al. 2011; Torres-Haro et al. 2021a; Visser et al. 2003). Alternatively, exposure to oxidative agents or phytohormones to increase astaxanthin production have been also investigated. Reactive oxygen species (ROS) generators such as ethanol (Yamane et al. 1997), antimycin A (Gil-Hwan et al. 1989), glutamate (Torres-Haro et al. 2021b; Wang et al. 2019), hydrogen peroxide (Ducrey-Santopietro et al. 1998; Gil-Hwan et al. 1989; Liu and Wu et al. 2006), and/or iron (Fe^2+^) (Bhosale 2004; Ducrey-Santopietro et al. 1998) induce *X. dendrorhous* cell damage and, as a response, the astaxanthin biosynthesis pathway is transcriptionally activated (Ana-Maria et al. 2022; Arunkumar et al. 2020; Domonkos et al. 2013; Ducrey-Santopietro et al. 1998; Yamane et al. 1997). However, high ROS concentrations may affect cell viability and, consequently, initial high astaxanthin production yields are transient (Gil-Hwan et al. 1989). On the other hand, phytohormones such as 6-benzylaminopurine (6-BAP) (Jun-Hui et al. 2020; Lu and Xu 2015; Pan et al. 2020) or gibberellic acid (Bu et al. 2020; Liu et al. 2022) shift the metabolic carbon flux from glycolysis towards amino acids, mevalonate and terpenoids biosynthetic pathways (Nutakor et al. 2022; Pan et al. 2017; Wang et al. 2019), which stimulates both biomass growth and astaxanthin synthesis without compromising cell viability. Because both oxidative agents and phytohormones stimulate astaxanthin production, but the latter may prevent the deleterious effect of the former since it promotes cellular resistance to reactive oxygen species, it is interesting to study their combined effect on astaxanthin biosynthesis.

In this study, astaxanthin biosynthesis in wild type *X. dendrorhous* (ATCC 24202) stimulated with glutamate, 6-BAP, indole-3-acetic acid (IAA), menadione, H_2_O_2_, and/or Fe^2+^, as a single or combined treatment (in several additions during cell growth and stationary phases), was evaluated, together with the transcriptional response of the astaxanthin biosynthesis pathway (*hmgR*, *idi*, *crtYB*, *crtR*, and *crtS*), the cellular viability and mitochondrial activity. Despite the independent effect of oxidative agents and phytohormones (6-BAP) on carotenoids synthesis in *X. dendrorhous* has been reported in other works (Ducrey-Santopietro et al. 1998; Pan et al. 2020), here, we report for the first time the effects of their combined use with the hypothesis that they activate the carotenoids synthesis in different ways and that phytohormones could mitigate the deleterious effect of oxidative agents. Our results indicated that an additive effect of 6-BAP and H_2_O_2_ (in several additions) reduces cell damage and promotes astaxanthin biosynthesis.

## Materials and methods

### Microorganism and culture medium

Wild type *X. dendrorhous* (ATCC 24202) was used in all the experiments. It was maintained on yeast/malt agar (YM) medium (3.0 g/L yeast extract, 3.0 g/L malt extract, 5.0 g/L peptone, 20 g/L glucose, and 20 g/L agar) and activated in a 250-mL flask containing 50 mL of a chemically defined (CD) mineral culture medium at pH 6.0 containing (g/L): 3.0, KH_2_PO_4_; 3.0, (NH_4_)_2_SO_4_; 3.0, Na_2_HPO_4_·12H_2_O; 0.1, glutamic acid; 20.0, glucose; 0.5, MgCl_2_·6H_2_O; 0.0192, ZnCl_2_; 0.0044, MnCl_2_·4H_2_O; 0.0005, CoCl_2_·6H_2_O; 0.0006, CuCl_2_·2H_2_O; 0.0003, (NH_4_)_6_Mo_7_O_4_·4H_2_O; 0.0174, CaCl_2_; 0.0116, FeCl_2_·4H_2_O; 0.003, H_3_BO_3_; 0.005, pantothenic acid; 0.005, nicotinic acid; 0.125, inositol; 0.005, thiamine; 0.005, pyridoxine; 0.001, *p*-aminobenzoic acid; and 0.000012, biotin. Nutrients were obtained from Sigma-Aldrich® (Saint Louis, Missouri, USA). Flask level cultures were incubated in a New Brunswick® Innova 44 rotary orbital (Hamburg, Germany) at 20 °C and 250 rpm (Farías-Álvarez et al. 2018; Tibayrenc et al. 2010; Torres-Haro et al. 2021b).

### Stimulation of carotenoids/astaxanthin production

In order to increase carotenoids/astaxanthin production, stimulating agents (50 mM, H_2_O_2_; 250 µM, Fe^2+^; 5 mM, menadione; 5.55 µM, 6-BAP; 50 µM, abscisic acid (ABA); and 220 µM, IAA) were individually supplemented to cultures during the exponential and stationary phases (5 applications at 24, 36, 48, 60, and 72 h of fermentation). Additional experiments were made using phytohormones (6-BAP, ABA, and IAA) in combination with H_2_O_2_ to evaluate the carotenoids/astaxanthin biosynthesis and to know their effect in the cell integrity and mitochondrial activity. Glutamate effect at high concentration (1.0 g/L) was also assayed and combined with oxidative agents and phytohormones. Fermentations were sampled just before and 2 h after each stimulation (Marcoleta et al. 2011) to evaluate gene expression (26, 38, 50, 62, and 74 h). All cultures were inoculated with 10% (v/v) pre-inoculum of the strain activated for 72 h in CD medium and incubated in a New Brunswick® Innova 44 rotary orbital (Hamburg, Germany) at 20 °C and 250 rpm (Farías-Álvarez et al. 2018; Tibayrenc et al. 2010; Torres-Haro et al. 2021b) during 120 h. Fermentations were carried out in duplicate.

### RNA isolation, single-strand DNA synthesis and RT-qPCR

Samples from fermentations supplemented with 6-BAP + H_2_O_2_ or ABA were collected and immediately frozen with liquid nitrogen and, subsequently, stored at − 80 °C until use. Total RNA extraction was performed with Tri Reagent® following the manufacturer instructions (Sigma-Aldrich®). The synthesis of the first-strand of complementary DNA (cDNA) was performed using SuperScript® III First-Strand Synthesis System (Thermo Fisher Scientific, Inc., Waltham, Massachusetts, USA) for RT-qPCR. The quality and quantity of the obtained cDNA were measured in a Thermo NanoDrop 1000 (Waltham, MA, USA) before RT-qPCR analysis. The relative transcript level for *hmgR*, *idi*, *crtYB*, *crtR*, and *crtS* were determined, using four biologic replicates, in a Rotor-GeneQ quantitative PCR system (QIAGEN N.V., Hilden, Germany). The differential gene expression was normalized to *X. dendrorhous* actin gene expression (Castelblanco-Matiz et al. 2015; Pan et al. 2020), and results were expressed as a function of the control using the 2^−ΔΔCT^ algorithm (Livak and Schmittgen 2001). Primers used (Supplemental Table S1) were designed in the ApE-A plasmid Editor software v3.0.8 (Davis and Jorgensen 2022).

### Analytical techniques

Biomass growth was determined by turbidimetry at 600 nm in an xMark™ spectrophotometer (Bio-Rad Laboratories, Inc., Irvine, CA, USA), and results were expressed as optical density (OD) units. Total carotenoid content was quantified in accordance with the methodology described by Sedmak et al. (1990). In brief, cells were disrupted using dimethyl sulfoxide and carotenoids were extracted using hexane–ethyl acetate mixture (1:1). Astaxanthin content was quantified using an HPLC (Agilent Technologies, Waldbronn, Germany) equipped with a UV–Vis detector and a Luna Phenomenex® column (particle size: 3 µm silica; pore size: 100 Å; dimensions: 15 × 4.6 mm) conditioned at 30 °C. A hexane–acetone mixture, 82:18 (v/v), was used as mobile phase at a flow of 1.2 mL/min (Torres-Haro et al. 2021b).

Cell viability and mitochondrial activity were determined by flow cytometry in a BD6 Sampler® (Becton, Dickinson and Company, Franklin Lakes, NJ, USA). Cell viability was assessed with 3,3′-dihexyloxacarbocyanine iodide (DiOC6) to measure membrane potential of viable cells, and propidium iodide (PI)-staining that binds to DNA of necrotic cells (Schulz-Hausmann et al. 2014). Mitochondrial activity was evaluated by MitoTracker®-staining (Baker et al. 2015), which passively diffuses across the plasma membrane and accumulates in active mitochondria.

### Statistical analysis

Analysis of variance (ANOVA) was performed with STATGRAPHICS® Centurion, Version 17 (Statgraphics Technologies, Inc., The Plains, Virginia, USA) for significant factors determination (*p*-value < 0.05).

## Results

### Effect of single stimulatory treatments on carotenoid biosynthesis

**Fig. 1:**
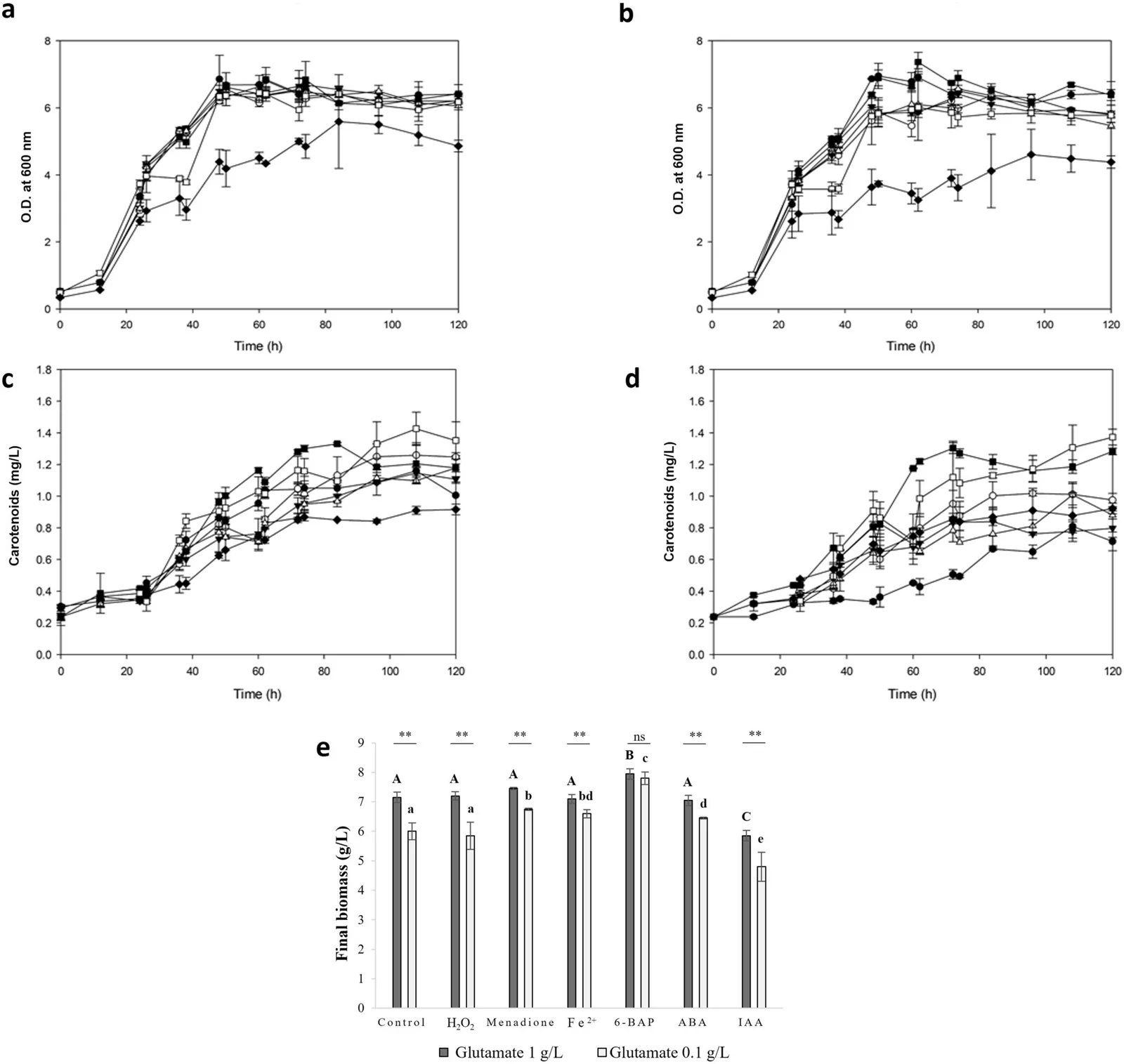
Stimulating agents effect on biomass growth and carotenoid production in *X. dendrorhous.* **a** Cell growth using a CD medium enriched with 1.0 g/L glutamate (  stimulated culture using only 1.0 g/L glutamate); **b** cell growth in a CD culture medium formulated at 0.1 g/L glutamate (  control treatment); **c** carotenoid production in a CD medium enriched with 1.0 g/L glutamate (  stimulated culture using only 1.0 g/L glutamate); **d** carotenoid production in a CD culture medium formulated at 0.1 g/L glutamate (  control treatment); **e** final biomass (g/L) of *X. dendrorhous* ATCC 24202 at 120 h fermentation. Lettering shows means that are significantly different (one-way statistical analysis; *p*-value ≤ 0.05) and ns indicate *p-*value ≥ 0.05. Capital letters designate comparisons using a CD medium enriched with glutamate, while lowercase letters designate comparisons using a CD medium at control conditions (glutamate 0.1 g/L). Moreover, one-way statistical analysis was performed to compare stimulating treatments using glutamate 1.0 or 0.1 g/L and ** indicates *p*-value ≤ 0.05.  H_2_O_2_,  menadione,  Fe^2+^,  6-BAP,  ABA or  IAA

In order to assess the individual effect of carotenoids/astaxanthin biosynthesis stimulating agents, each *X. dendrorhous* fermentation was supplemented 5 times along the cell growth and stationary phases with either oxidative agents: H_2_O_2_, Fe^2+^, menadione (Na et al. 2013), or phytohormones: 6-BAP, abscisic acid (ABA), or indole-3-acetic acid (IAA) (Jun-Hui et al. 2020; Liu et al. 2022; Lu and Xu 2015; Nutakor et al. 2022). Biomass growth (Fig. 1a and b), carotenoid production (Fig. 1c and d), final dry biomass (Fig. 1e), and yields (mg of carotenoids/g of biomass: *Y*_*P/X*_) were evaluated and compared to control treatment (Fig. 1d).

Cell growth and final biomass production were variable between stimulating treatments (Fig. 1a, b, and e). When H_2_O_2_ was added, no significant difference was observed in final biomass production compared to control (*p*-value > 0.05; Fig. 1e). However, when 6-BAP or ABA were added, an increase in final carotenoids production (0.53 ± 0.03 mg/L higher; *p*-value < 0.001) was observed compared to control (Fig. 1d). Nevertheless, cultures treated with IAA showed an inhibition of both cell growth and carotenoid production since the first addition at 24 h of fermentation (Fig. 1).

Additionally, glutamate has been reported as an activator of *gdh*, *idi*, and *crt* genes of the astaxanthin biosynthesis pathway, which push the carbon flux to carotenoids biosynthesis (Li et al. 2022; Wang et al. 2019). In order to assess the stimulatory glutamate effect on carotenoids biosynthesis in our working *X. dendrorhous* strain (ATCC 24202), growth kinetics was monitored for *X. dendrorhous* in a CD medium enriched with glutamate, 1.0 g/L (Fig. 1a), and compared to control treatment (using glutamate 0.1 g/L; Fig. 1b). An increase of the final biomass (1.19-fold higher; *p*-value < 0.05; Fig. 1a, b, and e) and the final carotenoid production were observed (1.43-fold higher;* p*-value < 0.05; Fig. 1c and d).

**Fig. 2:**
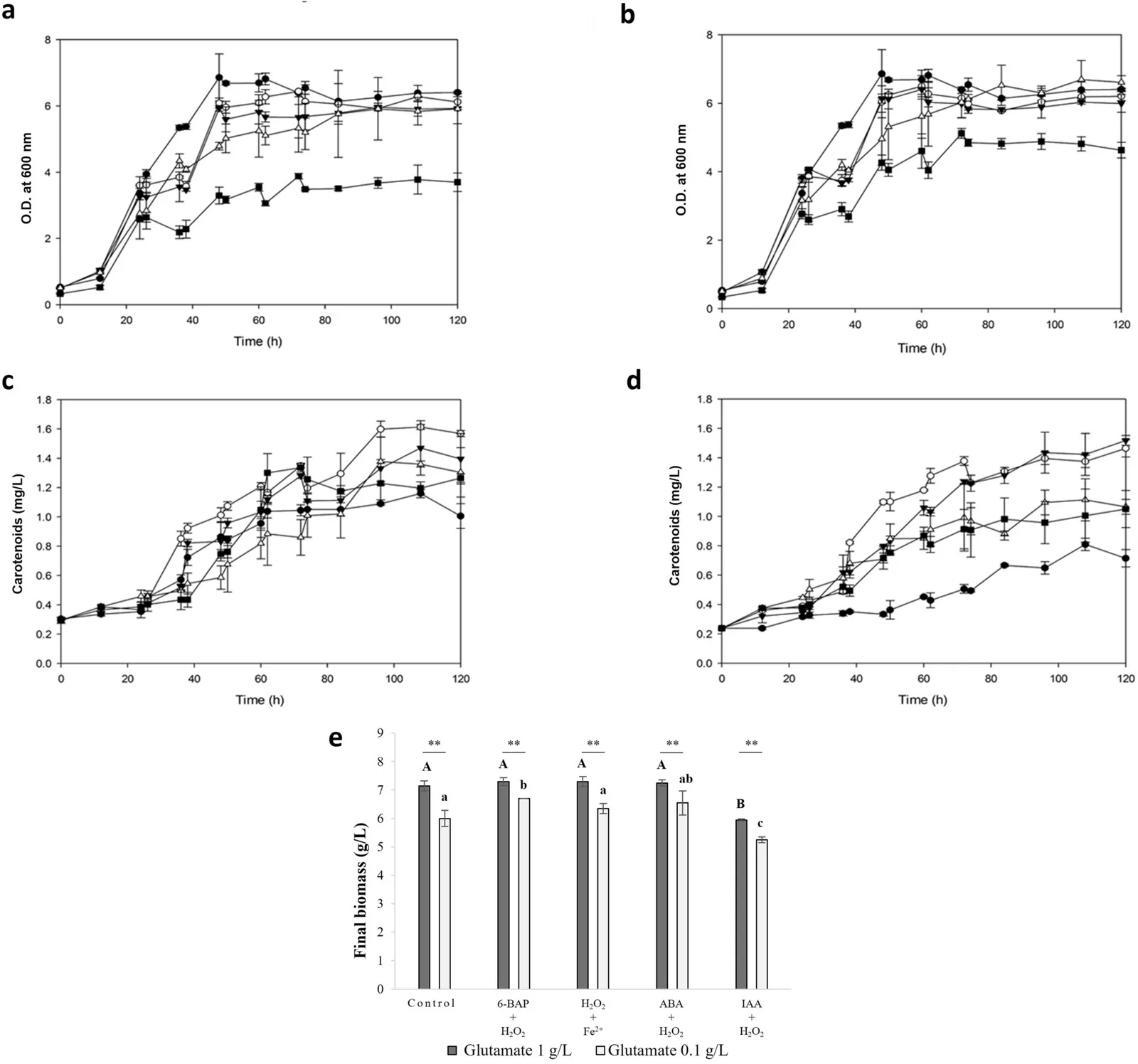
Effect of the combination of stimulating agents on biomass growth and carotenoid production in *X. dendrorhous.* **a** Cell growth in a CD medium enriched with 1.0 g/L glutamate (  stimulated culture using only 1.0 g/L glutamate); **b** cell growth in a CD culture medium formulated at 0.1 g/L glutamate (  control treatment); **c** carotenoid production in a CD medium enriched with 1.0 g/L glutamate (  stimulated culture using only 1.0 g/L glutamate); **d** carotenoid production in a CD culture medium formulated at 0.1 g/L glutamate (  control treatment); **e)** final biomass (g/L) of *X. dendrorhous* ATCC 24202 at 120 h fermentation. Lettering shows means that are significantly different (one-way statistical analysis; *p*-value ≤ 0.05). Capital letters designate comparisons using a CD medium enriched with glutamate, while lowercase letters designate comparisons using a CD medium at control conditions (glutamate 0.1 g/L). Moreover, one-way statistical analysis was performed to compared stimulated treatments using glutamate 1.0 or 0.1 g/L and ** indicates *p*-value ≤ 0.05.  6-BAP + H_2_O_2_,  H_2_O_2_ + Fe^2+^,  ABA + H_2_O_2_ or  IAA + H_2_O_2_

The stimulatory effect of oxidative agents and phytohormones was also analyzed using a CD medium enriched with glutamate, 1.0 g/L. An increase in final biomass production (7.0 to 7.95 g/L) after adding H_2_O_2_, menadione, Fe^2+^, or ABA was observed (*p*-value < 0.05; Fig. 1e) compared to control (6.0 ± 0.3 g/L) showing that stimulation did not lead to cell growth inhibition (Fig. 1a, b). However, the best treatment to increase biomass production (32.5%) was 6-BAP (Fig. 1e). Similarly, a stimulating effect on carotenoids biosynthesis was observed using H_2_O_2_, 6-BAP, or ABA (Fig. 2c), which increased 15.74, 23.15, and 31.48%, respectively, compared to control (final carotenoids production 0.81 mg/L). Moreover, those treatments also increased astaxanthin yields (Table 1).

**Table 1:** Astaxanthin biosynthesis stimulating agents evaluated on different *X. dendrorhous* strains

*X. dendrorhous* strain	Agent	Method	% Astaxanthin yield increase	Overexpressed genes	Reference
Wild type			0.17 to 0.2 mg/g (Basal level)		Domínguez-Bocanegra et al. (2007)
UV3-721		Carbon/nitrogen ratio (19:1)	22.1–22.4	*PDC1, idi, crtS*	Pan et al. (2017)
UV3-721	Glutamate (0.368 g/L)	Feeding at 24 h	40.7	*gdh, idi, crtI, crtR, crtS*	Wang et al. (2019)
AS 2.1557		Oxygen tension (25%)	61.6	*crtYB, crtI, crtR, crtS*	Wu et al. (2011)
ATCC 24202	Ethanol	pH–stat	62.5		Yamane et al. (1997)
UCD 67–385	Ethanol (2.0 g/L)	Feeding at 24 h	≈100	*crtYB, crtS*	Marcoleta et al. (2011)
25–2	Glutamate (1.0 g/L)	Carbon source	> 100		Torres-Haro et al. (2021b)
25–2	Glutamate (1.0 g/L)	Presence in the culture	14–25.6		Torres-Haro et al. (2021b)
JMU-MVP14	Glutamate (3.0 g/L)	Presence in the culture	30.9		Li et al. (2022)
JMU-MVP14	Fluconazol (120 mg/L)	Feeding at 48 h	25.8		Li et al. (2022)
PR106	Soybean oil (10 mL/L)	Presence in the culture	40		Li et al. (2023)
JMU‐MVP14	Sodium orthovanadate (2.5 mM)	Feeding in exponential and stationary phases	19.2	*crtE, crtI, crtYB, crtS*	Yang et al. (2023)
JMU‐MVP14	Melatonin (10 mM)	Feeding in exponential and stationary phases	30.3	*crtE, crtI, crtYB, crtS*	Yang et al. (2023)
67–210	Antimicin A	Feeding	> 0.4 mg/g		Gil-Hwan et al. (1989)
NRRL Y-17269	Hydrogen peroxide (0.01–0.1%)	Feeding	16	*ERG13*	Ducrey-Santopietro et al. (1998)
NRRL Y-17269	Fe^2+^ (450 µM)	Feeding	20		Ducrey-Santopietro et al. (1998)
NRRL Y-17269	H_2_O_2_ (0.1%) + Fe^2+^ (450 µM)	Feeding	30		Ducrey-Santopietro et al. (1998)
UV3-721	6-BAP (0.25 mg/L)	Feeding at 24 h	24.2	*hmgR, idi, crtE, crtYB, crtS*	Pan et al. (2020)
CBS 6938	Gibberellic acid (500 mg/L)	Feeding	50	ABC transporters, *crtS, gox, ldh*	Liu et al. (2022)
ATCC 24202	Glutamate (1.0 g/L)	Feeding in exponential and stationary phases	44.83*		This study
ATCC 24202	Glutamate (1.0 g/L) + H_2_O_2_ (50 mM)	Feeding in exponential and stationary phases	103.45*		This study
ATCC 24202	6-BAP (5.55 µM)	Feeding in exponential and stationary phases	124.14 ± 5.17*	*idi, crtYB, crtR, crtS*	This study
ATCC 24202	ABA (50 µM)	Feeding in exponential and stationary phases	160.34 ± 1.72*	*hmgR, idi, crtYB, crtR, crtS*	This study
ATCC 24202	Glutamate (1.0 g/L) + H_2_O_2_ (50 mM) + Fe^2+^ (250 µM)	Feeding in exponential and stationary phases	153.45*		This study
ATCC 24202	6-BAP (5.55 µM) + H_2_O_2_ (50 mM)	Feeding in exponential and stationary phases	268.97 ± 27.59*	*hmgR, idi, crtYB, crtR, crtS*	This study

*Increased yields (mg astaxanthin/g biomass: *Y*_*P*/*X*_) regarding a wild type strain in a low-cost CD mineral culture medium at 0.1 g/L of glutamate (control treatment)

The best single treatments to stimulate *X. dendrorhous* ATCC 24202 and obtain the highest biomass and carotenoids productions were 6-BAP and ABA. However, the maximal astaxanthin yields were obtained with ABA (0.20 to 0.23 mg of carotenoids/g of biomass: *Y*_*P/X*_; Table 1) using a CD medium enriched with glutamate or control conditions (glutamate 0.1 g/L; *p*-value > 0.05).

### Effect of combined stimulating agents on carotenoids/astaxanthin biosynthesis

**Fig. 3:**
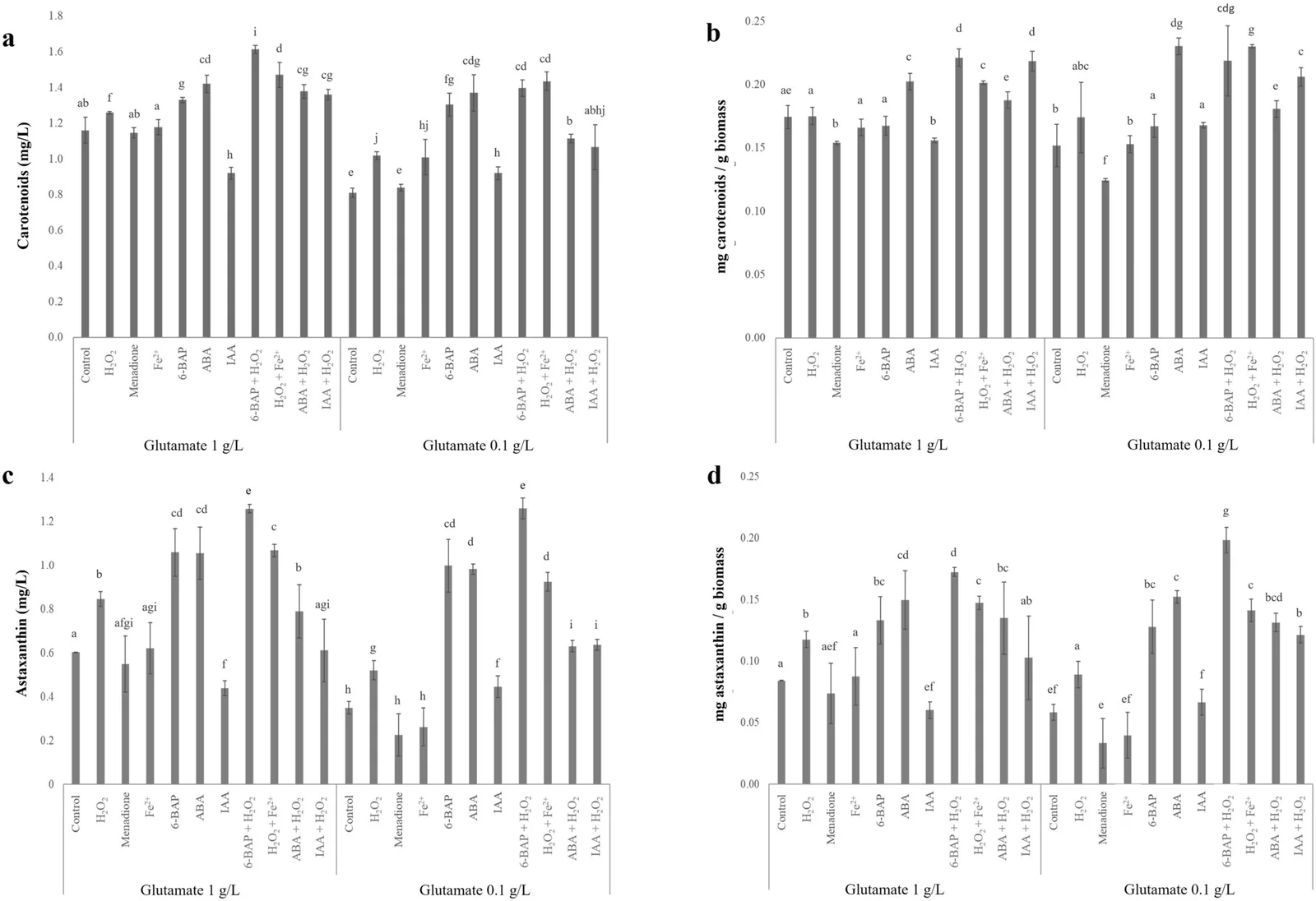
Comparison of treatments to stimulate the metabolic machinery involved on carotenoids and astaxanthin biosynthesis in *X. dendrorhous*. **a** Carotenoids production (mg/L) and **b** carotenoid yields (mg/g: mg of carotenoids/g of biomass; Y_P/X_). **c** Astaxanthin quantification (mg/L) by HPLC analysis for stimulating treatments. **d** Yields of astaxanthin production for stimulating treatments (mg/g: mg of astaxanthin/g of biomass). Results are shown as the mean with their standard deviation. Lettering shows means that are significantly different (one-way statistical analysis; *p*-value ≤ 0.05)

As previously mentioned, H_2_O_2_ using a CD medium enriched with glutamate increased carotenoids biosynthesis, while phytohormones stimulated both biomass and carotenoids production (Fig. 1; Table 1). Because of the latter, we hypothesized that oxidative agents and phytohormones stimulated the astaxanthin biosynthesis in different but complementary ways with potential interesting effects if used combined. Thus, phytohormones (6-BAP, ABA, and IAA) were selected as stimulating agents and evaluated in combination with H_2_O_2_ (phytohormone + H_2_O_2_) to promote high oxidative/metabolic stress, which, subsequently, over-activate the yeast defense metabolic machinery, the astaxanthin biosynthesis (Fig. 2). Moreover, in order to generate high levels of hydroxyl radicals, the reaction of H_2_O_2_ and Fe^2+^ (Fenton reaction) was evaluated on *X. dendrorhous*. Thereby, the cell growth (Fig. 2 a and b), carotenoid production (Fig. 2 c and d), and the final biomass (Fig. 2e) and carotenoid/astaxanthin yields (Fig. 3) were monitored for individual cultures of *X. dendrorhous* in a CD medium.

Stimulated cultures using 6-BAP + H_2_O_2_, H_2_O_2_ + Fe^2+^, or ABA + H_2_O_2_, using a CD medium enriched with glutamate, showed a biomass production between 7.15 to 7.30 g/L (above 20% higher compared to control using glutamate at 0.1 g/L; Fig. 2e). However, using 0.1 g/L glutamate and 6-BAP + H_2_O_2_, H_2_O_2_ + Fe^2+^, or ABA + H_2_O_2_, the biomass production was 6.5 ± 0.15 g/L (similar or even higher than the control; Fig. 2e). These results demonstrated the stimulating agents used in combination were not inhibitory to the cell growth; however, the carotenoid biosynthesis increased 80.24, 86.42, and 37.04% (between 1.37 to 1.61 mg carotenoids/L), respectively (Fig. 3a).

IAA + H_2_O_2_ treatment, similarly to the results obtained with single IAA treatment, led to a cell growth inhibition (Fig. 2b) and decreased the final biomass by 11.84% (Fig. 2e). However, the final carotenoid production increased 46.15% and 14.13% using a CD medium enriched with glutamate and in control conditions, respectively (Fig. 3a).

Combined treatments largely stimulated carotenoids biosynthesis compared to control treatment. Carotenoid production and carotenoid yield comparison, using individual or combined inductive treatments, are shown in Fig. 3a and b, respectively. Moreover, it is necessary to demonstrate the intracellular astaxanthin generated, as the greatest interest product in *X. dendrorhous*. Due to the above, the carotenoid extracts were analyzed by HPLC and astaxanthin quantification was performed. Astaxanthin production and astaxanthin yield comparison are shown in Fig. 3c and d. Combined 6-BAP and H_2_O_2_ was the best treatment to increase astaxanthin yields (Fig. 3d). The volumetric astaxanthin production increased 0.91 ± 0.01 mg/L, while astaxanthin yields increased 0.127 ± 0.018 mg/g biomass compared to the control treatment (*p*-value < 0.001; Fig. 3d; Table 1).

### *X. dendrorhous* viability and mitochondrial activity

Oxidative stress affects cell viability and, subsequently, astaxanthin production decreases (Arunkumar et al. 2020; Cortés-Rojo et al. 2009; Ducrey-Santopietro et al. 1998; Gil-Hwan et al. 1989; Laux and Nel 2001; Yamane et al. 1997). Therefore, it is important to finely regulate the oxidative/stimulating agents’ concentration to avoid negative cell affectations that hamper astaxanthin biosynthesis. In addition, mitochondrial activity could be compromised since oxidative/stimulating agents induce mitochondrial membrane damage, affecting components involved in the electron transport chain, which ultimately decreases ATP generation (Cao et al. 2020). Moreover, the isoprenoid synthesis (precursors of astaxanthin) occurs in the mitochondria (Cao et al. 2020; Ducrey-Santopietro et al. 1998; Torres-Haro et al. 2021a) and two important enzymes involved in astaxanthin biosynthesis are found in this organelle, acetyl-CoA acyltransferase, and cytochrome P450 reductase (EMBL-EBI 2022; Torres-Haro et al. 2021a).

Cell viability was determined by flow cytometry using PI and DiOC6 like cell staining at 24, 48, and 72 h (before stimulating agents’ addition), and 26, 50, and 74 h (after stimulating agents’ addition) in *X. dendrorhous* cultures during the exponential and stationary phases; results are shown in Table 2 and Supplemental Fig. S1. The mitochondrial activity was evaluated during exponential and stationary phases using MitoTracker® cell staining at 24 and 60 h (before stimulating agents addition), and 26 and 62 h (after stimulating agents addition), and results are shown in Table 3 and Supplemental Fig. S1. Evaluation at 62 h was assigned because at this time (just starting stationary phase), carotenoid production was still increasing (Figs. 1 and 2).

**Table 2:** Cell viability of stimulated *X. dendrorhous* cultures determined by flow cytometry using cell staining with PI and DiOC6

Treat ment	Time (h)											
	24				26				48			
Control	88.60	±	7.212	a	91.90	±	2.12	a	52.17	±	4.57	a
Glutamate 1.0 g/L	89.55	±	6.576	a	93.35	±	0.07	a	88.77	±	3.43	cd
H_2_O_2_	93.55	±	0.212	a	49.35	±	4.03	cde	58.15	±	4.03	a
Menadione	93.55	±	0.212	a	42.20	±	2.55	de	48.45	±	3.89	a
Fe^2+^	93.55	±	0.212	a	31.35	±	7.00	e	53.15	±	4.60	a
6-BAP	93.55	±	0.212	a	89.60	±	2.12	a	96.35	±	0.07	d
ABA	93.55	±	0.212	a	89.60	±	3.04	a	96.35	±	2.97	d
IAA	93.55	±	0.212	a	62.30	±	10.75	bcd	53.85	±	0.64	a
6-BAP + H_2_O_2_	93.55	±	0.212	a	72.20	±	0.00	ab	84.25	±	1.77	cd
H_2_O_2_ + Fe^2+^	93.55	±	0.212	a	86.65	±	0.21	a	75.60	±	9.05	bc
ABA + H_2_O_2_	93.55	±	0.212	a	58.35	±	2.47	bcd	57.65	±	7.85	a
IAA + H_2_O_2_	93.55	±	0.212	a	64.35	±	17.75	bc	63.20	±	3.25	ab
*LSD* p*-value	0.9374				0.0002				0.0000			

Treat ment												
	50				72				74			
Control	50.60	±	9.47	a	44.60	±	0.99	a	43.85	±	1.91	ab
Glutamate 1.0 g/L	85.90	±	3.68	de	83.05	±	5.30	cd	84.05	±	1.77	c
H_2_O_2_	68.60	±	5.66	bc	55.00	±	11.31	b	53.10	±	8.27	a
Menadione	45.00	±	2.55	a	40.85	±	1.20	a	53.10	±	3.25	a
Fe^2+^	49.85	±	4.60	a	44.35	±	1.34	a	52.95	±	5.59	a
6-BAP	96.75	±	1.63	e	95.35	±	0.07	d	93.10	±	2.83	c
ABA	96.75	±	2.47	e	95.35	±	3.39	d	93.10	±	3.32	c
IAA	49.40	±	7.50	a	22.30	±	1.98	a	21.80	±	5.37	d
6-BAP + H_2_O_2_	71.30	±	1.27	bc	78.70	±	2.55	bc	83.50	±	2.26	c
H_2_O_2_ + Fe^2+^	77.75	±	11.24	cd	83.80	±	3.25	c	84.55	±	0.21	c
ABA + H_2_O_2_	48.90	±	2.97	a	43.70	±	3.39	a	42.80	±	3.32	ab
IAA + H_2_O_2_	56.05	±	1.63	ab	40.25	±	2.33	ab	40.35	±	1.06	b
*LSD* p*-value	0.0001				0.0000				0.0000			

Results are shown as the mean with their standard deviation. Lettering shows means that are significantly different at same analysis time (one-way statistical analysis; *p*-value ≤ 0.05). *LSD least significant difference

**Table 3:** Mitochondrial activity of stimulated *X. dendrorhous* cultures determined by flow cytometry using cell staining with MitoTracker®

Treatment	Time (h)							
	24				26			
Control	82.80	±	0.92	a	84.00	±	1.20	ab
Glutamate 1.0 g/L	81.30	±	3.61	a	87.10	±	6.39	ab
H_2_O_2_	82.80	±	0.92	a	50.50	±	11.60	c
Menadione	82.80	±	0.92	a	50.50	±	1.70	c
Fe^2+^	82.80	±	0.92	a	50.80	±	1.56	c
6-BAP	82.80	±	0.92	a	87.60	±	0.21	ab
ABA	82.75	±	3.61	b	89.20	±	1.05	ab
IAA	82.75	±	0.92	b	76.40	±	0.70	a
6-BAP + H_2_O_2_	82.75	±	0.92	b	95.40	±	0.14	b
H_2_O_2_ + Fe^2+^	82.75	±	0.92	b	93.85	±	2.05	b
ABA + H_2_O_2_	82.75	±	0.92	b	90.90	±	1.84	ab
IAA + H_2_O_2_	82.75	±	0.92	b	87.05	±	0.07	ab
*LSD* p*-value	0.0003				0.0000			

Treatment								
	60				62			
Control	88.80	±	3.43	ab	85.30	±	2.97	a
Glutamate 1.0 g/L	88.77	±	3.43	ab	85.90	±	3.68	ab
H_2_O_2_	58.15	±	0.57	c	68.60	±	1.41	f
Menadione	48.45	±	0.85	d	45.00	±	0.14	e
Fe^2+^	53.15	±	0.00	cd	49.85	±	1.63	e
6-BAP	96.35	±	7.78	a	96.75	±	3.18	cd
ABA	85.60	±	0.58	b	87.85	±	1.17	ab
IAA	92.25	±	0.14	ab	91.20	±	0.42	bc
6-BAP + H_2_O_2_	97.00	±	0.71	a	97.00	±	0.42	d
H_2_O_2_ + Fe^2+^	96.30	±	0.85	a	95.40	±	1.13	cd
ABA + H_2_O_2_	96.45	±	0.49	a	95.60	±	1.13	cd
IAA + H_2_O_2_	96.70	±	0.14	a	95.60	±	0.42	cd
*LSD* p*-value	0.0000				0.0000			

Results are shown as the mean with their standard deviation. Lettering shows means that are significantly different at same analysis time (one-way statistical analysis; *p*-value ≤ 0.05). *LSD: least significant difference

Before the stimulating agents addition at 24 h fermentation, cell viability and mitochondrial activity were around 90 and 80%, respectively (Supplemental Fig. S1). Then, 2 h after the first addition of H_2_O_2_, Fe^2+^, menadione, IAA, IAA + H_2_O_2_, and ABA + H_2_O_2_ (oxidant/stimulating agents), a significative decrease on cell viability was observed (< 64.35%; Table 2). A similar decrease was observed in the mitochondrial activity after H_2_O_2_, Fe^2+^, or menadione additions (< 58.15% at 26 h; Table 3). Nevertheless, using a CD medium enriched with glutamate after to use oxidant agents such as stimulating, cell viability and mitochondrial activity could be recuperated (Supplemental Fig. S1).

Employing 6-BAP or ABA as stimulating agents, no effect in cell viability (> 90%; Table 2; Supplemental Fig. S1) or mitochondrial activity (> 82.75%; Table 3; Supplemental Fig. S1) was observed, even in stationary phase fermentation. Also, using 6-BAP + H_2_O_2_ did not compromise cell viability, maintaining it above 70% during the fermentation process (Table 2; Supplemental Fig. S1).

### Transcriptional response of genes encoding for carotenoids biosynthesis

**Fig. 4:**
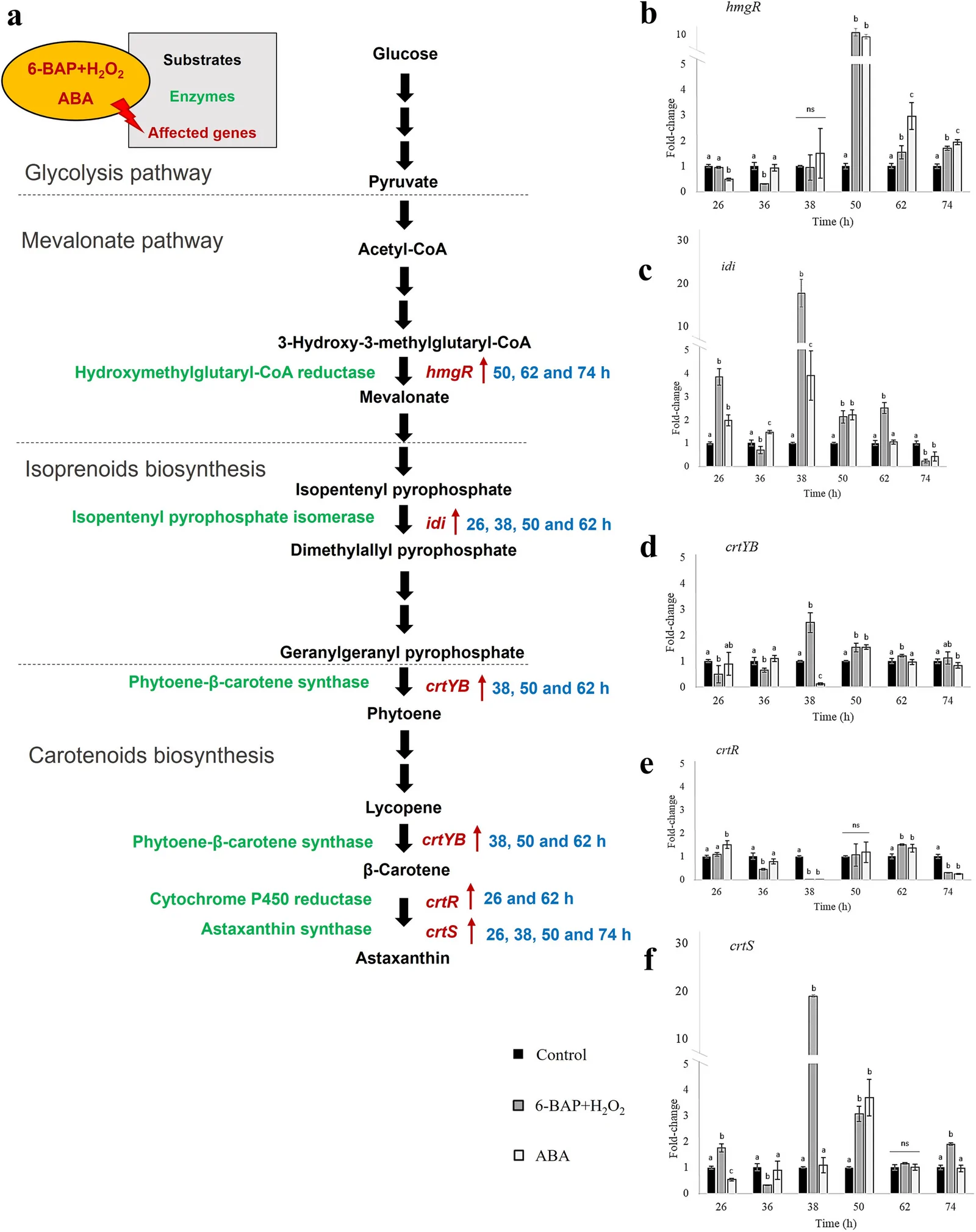
RT-qPCR expression analysis of *X. dendrorhous* ATCC 24202 genes involved in carotenoids/astaxanthin biosynthesis stimulated by 6-BAP and H_2_O_2_ (combined treatment), and ABA. **a** General scheme of astaxanthin biosynthesis pathway. **b** *hmgR* expression levels, **c** *idi* expression levels, **d** *crtYB* expression levels, **e** *crtR* expression levels, and **e** *crtS* expression levels. Analysis point times at 26, 38, 50, 62, and 74 h (after stimulation). Additionally, time 36 h was evaluated, just before second stimulation to demonstrated that transcriptional responses such as *hmgR* or *crt* family genes decrease through time. Each transcriptional level was normalized to the expression of the housekeeping actin gene and expressed as a function of the control treatment (relative expression = 1; black bars). Lettering shows means that are significantly different at evaluated time (one-way statistical analysis; *p*-value ≤ 0.05) and ns indicate *p-*value ≥ 0.05. The values are the mean of four independent experiments, and the error bars correspond to the standard deviations. *hmgR*, hydroxymethylglutaryl-CoA reductase; *idi*, isopentenyl pyrophosphate isomerase; *crtYB*, phytoene-β-carotene synthase; *crtR*, cytochrome P450 reductase; *crtS*, astaxanthin synthase

*Xanthophyllomyces dendrorhous* cultures stimulated using ABA or 6-BAP + H_2_O_2_ (the best treatments using a CD medium enriched with glutamate or control conditions; Fig. 3) increased the metabolic carbon flux towards astaxanthin biosynthesis (Fig. 4a). Thereby, the relative expression levels of *hmgR* (Fig. 4b), *idi* (Fig. 4c), *crtYB* (Fig. 4d), *crtR* (Fig. 4e), and *crtS* (Fig. 4f) were measured (all of them involved in the carotenoid biosynthesis pathway; Fig. 4a). Expression levels were determined at 26, 38, 50, 62, and 74 h fermentation (after the stimulations) and expressed as a function of a control treatment compared to ABA or 6-BAP + H_2_O_2_.

During the first stimulation of *X. dendrorhous* sampled at 26 h using 6-BAP + H_2_O_2_, *idi* and *crtS* expression levels increased 3.83 and 1.76-fold, respectively, demonstrating that the yeast generated a positive response to stimulate the astaxanthin biosynthesis pathway. However, after 10 h without stimulation, gene expression decreased to levels below the control treatment. During the second stimulation at 38 h with 6-BAP + H_2_O_2_, *idi*, *crtYB*, and *crtS* expression levels increased 22.24, 2.49, and 21.93-fold, respectively, while *crtR* and *hmgR* showed lower expression levels compared to the control treatment. During the third stimulation at 50 h, the yeast generated a new response: *idi*, *crtYB*, and *crtS* expression levels decreased to 2.12, 1.53, and 3.07-fold, respectively, while *hmgR* increased to 9.73-fold and *crtR* maintained similar levels to that of the control treatment (1.06-fold). During the fourth stimulation at 62 h, *hmgR*, *idi*, and *crtR* were overexpressed 1.54, 2.50, and 1.50-fold, respectively, while *crtYB* and *crtS* showed expression levels similar to that of the control (1.2 and 1.15-fold, respectively). Finally, during the last stimulation performed (fifth stimulation at 74 h), *hmgR* and *crtS* were overexpressed 1.70 and 1.90-fold, respectively; *crtYB* was similar to the control, but *idi* and *crtR* were upregulated comparing to the control treatment (Fig. 4).

Transcriptional responses to ABA (Fig. 4). During the first stimulation sampled at 26 h, *idi* expression levels increased 1.97-fold, demonstrating that the yeast generated a positive response since the stimulation process began. Unlike the 6-BAP + H_2_O_2_ effect, it was observed that after 10 h without stimulation, the expression levels of *idi* were maintained (1.47-fold). During the second stimulation at 38 h, *hmgR* and *idi* expression levels increased 1.5 and 3.89-fold, respectively, while *crtYB* and *crtR* were downregulated, and *crtS* showed similar expression levels compared to the control treatment (1.09-fold). During the third stimulation at 50 h, similar to 6-BAP + H_2_O_2_ effect, the yeast increased *idi*, *crtYB*, and *crtS* expression levels to 2.21, 1.54, and 3.70-fold, respectively, while *hmgR* increased to 9.30-fold and *crtR* maintained similar levels compared to the control treatment (1.18-fold). During the fourth stimulation at 62 h, *hmgR* and *crtR* were overexpressed 2.96 and 1.36-fold, respectively, while *idi*, *crtYB*, and *crtS* showed expression levels similar to the control (1.04, 0.96, and 1.01-fold, respectively). Finally, during the last stimulation performed (fifth stimulation at 74 h), *hmgR* was overexpressed 1.94-fold, *crtYB* and *crtS* remained similar to the control, but *idi* and *crtR* were upregulated compared to control.

To assess the individual response to 6-BAP and H_2_O_2,_ the components were individually added on *X. dendrorhous* and, subsequently, transcriptional response was analyzed in RT-qPCR at 38 h during the exponential growth phase (sampled after the second stimulation, where the yeast generates a major response in carotenoids production velocity). 6-BAP decreased the *hmgR* expression levels but increased those of *idi*, *crtYB*, *crtR*, and *crtS* (2.54, 3.55, 25.95, and 2.77-fold, respectively), compared to control treatment (Supplemental Fig. S2). No significant difference was observed using 6-BAP in *crtYB* expression levels (*p*-value > 0.05). Using H_2_O_2_, gene expression decreases at 38 h of fermentation (Supplemental Fig. S2).

## Discussion

In this study, the effective stimulation of the astaxanthin pathway of wild type *X. dendrorhous* (ATCC 24202) (*hmgR, idi*, *crtYB*, *crtR*, and *crtS* responses), as well its cellular integrity, were evaluated (Table 2; Supplemental Fig. S1). Stimulating agents such as glutamate, H_2_O_2_, 6-BAP, and/or ABA significantly affected biomass production, carotenoids/astaxanthin biosynthesis, and the stress molecular responses (Fig. 4). Employing these agents in temporally spaced additions on *X. dendrorhous*, a positive stimulating effect on astaxanthin biosynthesis was observed compared to the control treatment (Fig. 3).

Glutamate has been reported as an essential nutrient for *X. dendrorhous* cultures, which increases the metabolic carbon flux towards astaxanthin biosynthesis by over-activating the expression of *gdh*, *idi*, *crtI*, *crtR*, and *crtS*. Astaxanthin overproduction in *X. dendrorhous* UV3-721 at 24 h after glutamate addition increased astaxanthin yields by 44.7% (Wang et al. 2019). In this study, using a CD medium enriched with glutamate (1.0 g/L), astaxanthin yields increased 44.83% compared to control (Fig. 3d), similar to that obtained by UV3-721 mutant strain (Wang et al. 2019). In addition, using glutamate (> 0.1 or, specifically, 1.0 g/L), it is possible to maintain *X. dendrorhous* cell integrity (Table 2; Supplemental Fig. S1) and increase biomass production (1.15 g/L) and astaxanthin yields (0.026 mg/g biomass).

Using a CD medium enriched with glutamate and combined with H_2_O_2_, 6-BAP or ABA increased the stimulating effect on astaxanthin biosynthesis (Fig. 3). However, ABA or 6-BAP, using both a CD medium enriched with glutamate or control conditions, were the best individual treatments to stimulate astaxanthin biosynthesis (1.24- and 1.60-fold, respectively; Fig. 3c). Those results show the importance not only of the stimulation process; also, it is necessary to add nutrients to help the cell to support the stress generated.

It has been reported that the H_2_O_2_ (between 2 and 10 mM) and/or Fe^2+^ (between 0.45 and 0.9 mM) generate hydroxyl radicals that lead to an increase on carotenoids production in *X. dendrorhous* as a response to oxidative stress (Gil-Hwan et al. 1989; Bhosale 2004; Ducrey-Santopietro et al. 1998; Cortés-Rojo et al. 2009). H_2_O_2_ stimulates the sterol biosynthesis pathway that is directly linked to carotenoid biosynthesis, starting with the hydroxy-methyl-glutaryl-CoA (HMG-CoA) production through *ERG13* overexpression (Gil-Hwan et al. 1989). It also induces oxidative stress in mitochondria leading to increased catalase (CAD), superoxide dismutase (SOD), and glutathione peroxidase (GSH-Px) activities; additionally, the cell activates the expression of the *crt* gene family and carotenoids/astaxanthin biosynthesis (Guo-Liang et al. 2011; Liu and Wu 2006; Reyes et al. 2014; Wang et al. 2019). However, high oxidative stress may affect the cell integrity, which leads to a decrease on astaxanthin biosynthesis (Fig. 3). Therefore, yeasts adapted to high oxidative stress (using H_2_O_2_) have been used to stimulate carotenoids production up to 18 mg/g of dry biomass (Reyes et al. 2014).

In this study, wild type *X. dendrorhous* (ATCC 24202) growth, after adding H_2_O_2_, menadione, or Fe^2+^ as stimulating agents, was inhibited by 1.31 ± 0.17 OD units compared to control treatment (Fig. 1b). The same effect was observed in cell viability and mitochondrial activity, where both decreased > 50.50% during the first stimulation and cells did not return to the basal condition (Table 2 and 3). Using H_2_O_2_, menadione, and Fe^2+^, astaxanthin production levels and astaxanthin yields decreased > 0.30 mg/L (Fig. 3c) and > 0.03 mg/g (Fig. 3d), respectively, compared to the same conditions using a CD medium enriched with glutamate. These results indicated that high cellular oxidative stress does not stimulate astaxanthin biosynthesis (Fig. 3) and, probably, CAD, SOD, and GSH-Px activities are not enough to avoid mitochondrial/cell damage (Table 2). However, using a CD medium enriched with glutamate, in addition to generate a higher stimulating effect on carotenoids/astaxanthin production, also activates the defense mechanisms (protector effect on cell integrity is maintained) in non-adapted strains to oxidative stress conditions (Table 2; Supplemental Fig. S1).

In addition to oxidative stress agents, phytohormones have been reported as attractive astaxanthin stimulating agents (Bu et al. 2020; Jun-Hui et al. 2020; Liu et al. 2022; Lu and Xu 2015; Nutakor et al. 2022; Pan et al. 2020). IAA has been reported as a powerful auxin capable of stimulate the cell growth and carotenoids production in microalgae such as *Chromochloris* sp., *Chlamydomonas* sp*.*, *Chlorella* sp., or *H. pluvialis* (Jun-Hui et al. 2020; Lu and Xu 2015). Specifically, in *Chromochloris zofingiensis* using IAA at concentrations between 37.8 and 94.58 µM, biomass production increased 26.7%, and astaxanthin production > 22% (Jun-Hui et al. 2020). Mechanistically, IAA is molecularly recognized by auxin receptors (auxin transcription factors) such as PIN3, TIR1, AFB, and ARFs and, subsequently, they stimulate the expression of enzymes involved in lipids, sugars, and/or ROS biosynthesis that activate carotenoids/astaxanthin biosynthesis response (Jun-Hui et al. 2020; Lu and Xu 2015). However, the transcription factors PIN3, TIR1, AFB, and ARFs are not present in *X. dendrorhous* (EMBL-EBI 2022; UniProt 2022) and, then, astaxanthin production was not affected compared to control treatment (Fig. 3c). Probably, IAA could be a toxic agent to yeasts, as suggested its effect in treatments where IAA was added as a stimulating agent (Fig. 1e; Table 2). However, it is unknown how this auxin induces inhibition on *X. dendrorhous*.

6-BAP acts like a metabolic stressor, inhibits the tricarboxylic acid cycle, and increases the carbon/nitrogen flux assimilation toward carotenoids biosynthesis (Pan et al. 2020). Due to 6-BAP metabolic effect, it is not necessary to saturate tricarboxylic acid cycle using a culture medium enriched with glutamate (Fig. 3c) to displace the carbon flux towards carotenoids/astaxanthin biosynthesis (Wang et al. 2019). In *X. dendrorhous*, microalgae or plants (Jun-Hui et al. 2020; Lu and Xu 2015; Nutakor et al. 2022; Pan et al. 2020; Torres-Haro et al. 2021a), 6-BAP activate histidine kinase (AHK) and histidine phosphotransferase (AHP) (EMBL-EBI 2022; Jun-Hui et al. 2020; Lu and Xu 2015) and, subsequently, they activate the expression of enzymes involved in amino acids, lipids, and carotenoids/astaxanthin biosynthesis (Fig. 4a). In addition, it has been reported that 6-BAP, during the exponential and stationary phases, increases *hmgR*, *idi*, *crtE*, *crtYB*, and *crtS* expression levels that are directly involved in the carotenoid and astaxanthin biosynthesis pathway in *X. dendrorhous* (Pan et al. 2020; Nutakor et al. 2022). Transcriptional profiles determined in this study at 38 h on *X. dendrorhous* (cell growth exponential phase, sampled during the second stimulation) increased the *idi*, *crtYB*, *crtR*, and *crtS* expression levels (Supplemental Fig. S2). Gene expression was different only in *crtR*, which means the cytochrome P450 reductase is also involved in astaxanthin stimulation using 6-BAP.

The ABA effect has been studied on biomass growth, stress tolerance, tolerance to osmotic pressure, high salinity, and induction of carotenoid biosynthesis, in different microalgae such as *H. pluvialis*, *Chlamydomonas* sp., *Nannochloropsis oceanica*, *Dunaliella* sp., and *C. zofingiensis* (Jun-Hui et al. 2020; Lu and Xu 2015). ABA has not been used neither to stimulate astaxanthin biosynthesis in *X. dendrorhous*, nor to evaluate its effect upon transcriptional responses, and cell integrity; those effects were assessed for the first time in the present study (Fig. 4). ABA was the best individual treatment to increase astaxanthin yields and maintain the cell integrity. However, combined treatment of ABA + H_2_O_2_ resulted in a minor astaxanthin biosynthesis stimulation and generated cell damage (42.80% cell viability at 74 h; Table 2). The main receptors for ABA are APC (anaphase promoting complex) and phosphatases (PP2C) which are present in the *X. dendrorhous* (EMBL-EBI 2022; UniProt 2022). These proteins are known to activate the response of serine/threonine-protein kinase and mitogen-activated protein kinase indicating the cell the presence of genotoxic stresses such as ionizing radiation, UV light, or DNA replication stalling; thereby, it acts as a DNA damage sensor that induces a defense response such as the carotenoid and/or astaxanthin biosynthesis. Probably, combined ABA + H_2_O_2_ treatment resulted in a high oxidative stress signals and, consequently, decreased carotenoids/astaxanthin biosynthesis stimulation (Fig. 3).

Transcriptional ABA responses (Fig. 4) were demonstrated since the first stimulation at 26 h, increasing *idi* expression, nevertheless, cell growth inhibition was observed (Fig. 1b). However, at 36 h (before the second stimulation), an increase of 0.16 mg/L on carotenoids production using this phytohormone was observed indicating an astaxanthin induction in surviving yeast cells (Fig. 3). After the second stimulation (38 h), *idi* expression increased. This transcriptional response and carotenoids production allowed the yeast to maintain a low oxidative stress avoiding the growth inhibition phase, and increasing DO (Fig. 1b). Moreover, during the stationary phase for ABA treatment (after 48 h; Fig. 1b), in addition to *idi*, it was observed an increase on transcriptional responses for *hmgR*, *crtYB*, *crtR*, and *crtS* (Fig. 4) and, at same time, increased carotenoids production (> 0.5 mg/L; Fig. 1d) and maintained *X. dendrorhous* cell integrity (Table 2; Supplemental Fig. S1). Those results show that ABA generates a cell growth inhibition, but after the yeast surpasses a new ABA addition, *crt* gene expression and astaxanthin production increased. The *crt* family gene overexpression levels and astaxanthin production have been reported for mutant strains in exponential and/or stationary phases (Castelblanco-Matiz et al. 2015; Contreras et al. 2013; Pan et al. 2020; Wang et al. 2019). However, as observed in this study, the stimulating effect of ABA and the transcriptional responses that produces on wild type strains is temporary (Fig. 4) and it is necessary to add more than one addition to obtain the stimulation of astaxanthin biosynthesis pathway.

Combined treatment of 6-BAP + H_2_O_2_ was the best potential alternative to increase carotenoid biosynthesis (Fig. 3a) and astaxanthin yields (Table 1). During the first stimulation at 26 h using these stimulating agents, cell viability decreased 21.35% (Table 2), but a high mitochondrial activity remained (95.40%). *idi* and *crtS* expression levels increase 3.84- and 1.76-fold (Fig. 4). The cell response to the cell damage for the stress increased carotenoid production (Fig. 2d). However, cell growth inhibition was observed at 36 h (Fig. 1b) but carotenoid production increased 0.15 mg/L compared to control even though the positive observed transcriptional response had disappeared (Fig. 4). After the second stimulation (38 h), increased *idi* and *crtS* expression levels (Fig. 4) and, probably, there are other transcriptional responses that allow it to maintain/adapt to high oxidative stress avoiding the cell growth inhibition (Fig. 1b), increasing DO (Fig. 2b), and cell viability at 48 h (Table 2). During stationary phase, at third stimulation (after 48 h), in addition to *idi* and *crtS*, an increased *hmgR* (similar to ABA treatment) and *crtYB* and, at same time, increased carotenoid production 0.74 mg/L compared to control (Fig. 2d). This effect is due to a high cell integrity (> 72.20%) compared to control (Table 2). At 74 h, during the fourth stimulation and reaching the maximum carotenoids production (Fig. 2b), cell viability increased to 83.50% (Table 2) and there was an increase on transcriptional responses for *hmgR*, *idi*, *crtYB*, *crtR*, and *crtS* (Fig. 4). The transcriptional responses and high cellular integrity allowed the yeast to generate 0.88 mg carotenoids/L higher compared to the control at this time. Finally, at 84 h of the growth during the fifth stimulation, overexpression of *hmgR* and *crtS* (1.70- and 1.90-fold, respectively) was observed, maintaining high mitochondrial activity (97.0%; Table 3) and increasing carotenoid production (Fig. 3c).

The 6-BAP + H_2_O_2_ treatment, similar to plants and/or microalgae (Jun-Hui et al. 2020; Lu and Xu 2015), allows higher transcriptional responses for the damage caused by oxidative stress (H_2_O_2_ effect) and inhibition compounds stress (6-BAP addition) that leads to the expected cellular response, the stimulation of carotenoid/astaxanthin biosynthesis. The cell damage probably is minimal and allows to *X. dendrorhous* the energy generation to maintain the cell growth since it had a protective effect on mitochondria during the fermentation and its activity was highest compared to control (increased 14.25%; Table 3).

In conclusion, phytohormones such as 6-BAP, ABA or 6-BAP + H_2_O_2_ can be stimulating treatments for increased astaxanthin biosynthesis in wild type *X. dendrorhous* without compromising cellular integrity compared to other oxidative/stimulating agents, reported for the first time in this study. However, 6-BAP + H_2_O_2_ increased transcriptional signals compared to ABA and promoted a higher yeast response, astaxanthin biosynthesis. Moreover, stimulation reinforcements (several additions of stimulating agents during the exponential and stationary phases) are necessary since transcriptional responses such as *hmgR*, *idi*, or *crt* family genes decrease after 12 h of fermentation (Fig. 4).

The astaxanthin biosynthesis stimulation represents a potential alternative capable of allowing to elucidate other genes (direct/indirect or turn on/off) within the metabolic machinery of *X. dendrorhous* and, later, lead to establish a metabolic engineering strategy that allows the astaxanthin overproduction without compromising cellular integrity. In this study, it was demonstrated that additionally to *crt* family genes, *hmgR* and *idi* could be specific targets in metabolic engineering techniques on *X. dendrorhous*.

## Supplementary Information

Below is the link to the electronic supplementary material.

Supplementary file1 (PDF 1058 kb)

## Data Availability

All data generated or analyzed during this study are included in this published article and its supplementary information files.

## Declarations

### Disclaimer

The funders had no role in the design of the study; in the collection, analyses, or interpretation of data; in the writing of the manuscript; or in the decision to publish the results.
