# Supplementary material for: Combined 6-benzylaminopurine and H2O2 stimulate the astaxanthin biosynthesis in Xanthophyllomyces dendrorhous
Source: Appl Microbiol Biotechnol. 2024 Jan 22;108(1):158. doi: 10.1007/s00253-023-12875-9 (PMC10803577; doi:10.1007/s00253-023-12875-9)
Supplement: Supplementary file 1 — Supplementary file1 (PDF 1058 kb) [file 253_2023_12875_MOESM1_ESM.pdf]

Applied Microbiology and Biotechnology

COMBINED 6-BENZYLAMINOPURINE AND H_2O_2 STIMULATE THE ASTAXANTHIN BIOSYNTHESIS IN *Xanthophyllomyces dendrorhous*

Alejandro Torres-Haro, Jorge Verdín, Manuel R. Kirchmayr, Melchor Arellano-Plaza*

Biotecnología Industrial, Centro de Investigación y Asistencia en Tecnología y Diseño del Estado de Jalisco, A.C. Camino Arenero 1227, El Bajío del Arenal, Zapopan, Jalisco, Mexico, 45019.

***Corresponding author:** Melchor Arellano-Plaza

Centro de Investigación y Asistencia en Tecnología y Diseño del Estado de Jalisco, A. C. Camino Arenero 1227, Col. El Bajío del Arenal, C.P. 45019, Zapopan, Jalisco, Mexico.

Tel.: +52 (33) 33455200 Ext. 1313

e-mail: marellano@ciatej.mx

Here, is presented the supplemental material:

Supplemental Tables

Supplemental Table S1. Mature transcript gene-specific primers used for RT-qPCR analysis in this study.

Primer name*	Sequence (5´-3´)	Target gene cDNA	Source
mact-F	CCGCCCTCGTGATTGATAAC	*actin*	Casteblanco-Matiz et al. 2015
mact-R	TCACCAACGTAGGAGTCCTT	*actin*	Casteblanco-Matiz et al. 2015
mmhmgR-F	CATTGATGGAGGCTGTGAACG	*hmgR*	This study
mmhmgR-R	GTCGATCGATTGTGCTTCATGTG	*hmgR*	This study
mmidi-F	GAGTACGATGAGGAGCAGGTCAG	*idi*	This study
mmidi-R	GTCGAACACCGATCTGGTTC	*idi*	This study
mmcrtYB-F	AGACAGCGGAAGAATACCGAC	*crtYB*	This study
mmcrtYB-R	GAACAAACGATGGAAAGGAGGA	*crtYB*	This study
mmcrtR-F	CTGGGAAACAAGACCTACGA	*crtR*	This study
mmcrtR-R	GGAACCTCGGTTACGACAAA	*crtR*	This study
mmcrtS-F	ATGGCTCTTGCAGGGTTTGA	*crtS*	Casteblanco-Matiz et al. 2015
mmcrtS-R	TGCTCCATAAGCTCGATCCCAA	*crtS*	Casteblanco-Matiz et al. 2015

mm: mature transcript
*F (forward) and R (reverse) in the primer name indicate the primer orientation.

Supplemental Figures

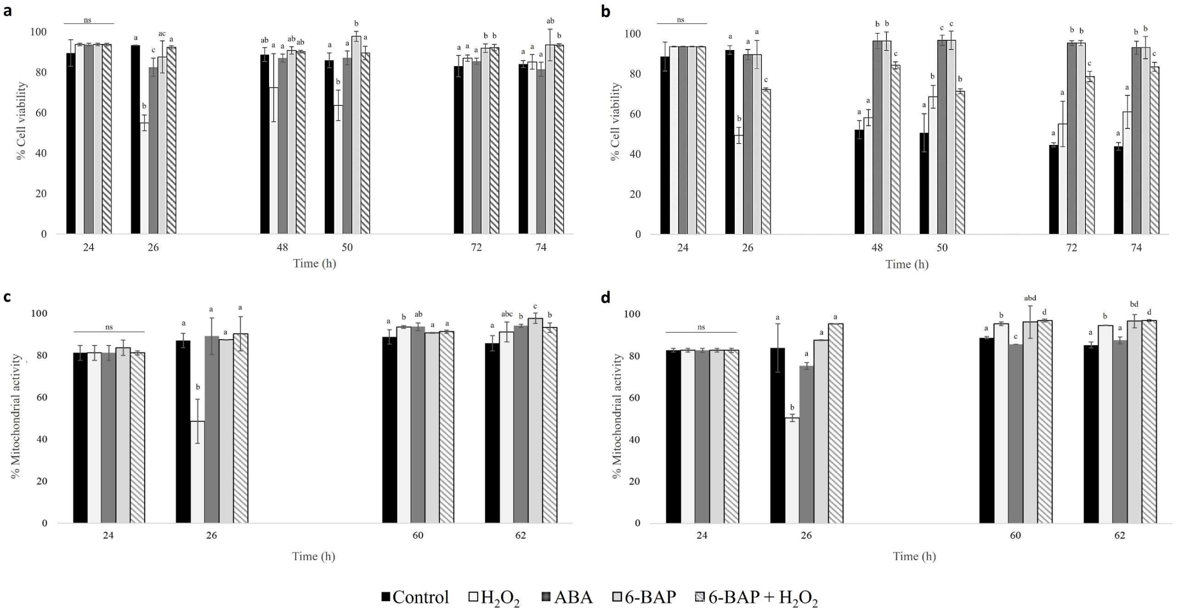

Supplemental Fig. S1. Cell viability and mitochondrial activity of stimulated *X. dendrorhous* cultures determined by flow cytometry. **a)** Cell viability of stimulated *X. dendrorhous* grown in a CD medium enriched with glutamate, **b)** cell viability of stimulated *X. dendrorhous* grown in CD culture medium formulated with 0.1 g/L glutamate (control concentration). Cell viability was evaluated times before stimulating agents addition: 24, 48 and 72 h, and, subsequently, at evaluated times after stimulating agents addition at 26, 50 and 74 h. **c)** Mitochondrial activity in stimulated *X. dendrorhous* grown in a CD medium enriched with glutamate, and **d)** mitochondrial activity for stimulating agents in a CD culture medium formulated at 0.1 g/L of glutamate (control concentration). Mitochondrial activity was evaluated times before stimulating agents addition: 24 and 60 h, and, subsequently, at evaluated times after stimulating agents addition at 26 and 62 h. Lettering shows means that data are significantly different at evaluated time (one-way statistical analysis; p-value≤0.05) and ns (not significant) indicate p-value≥0.05.

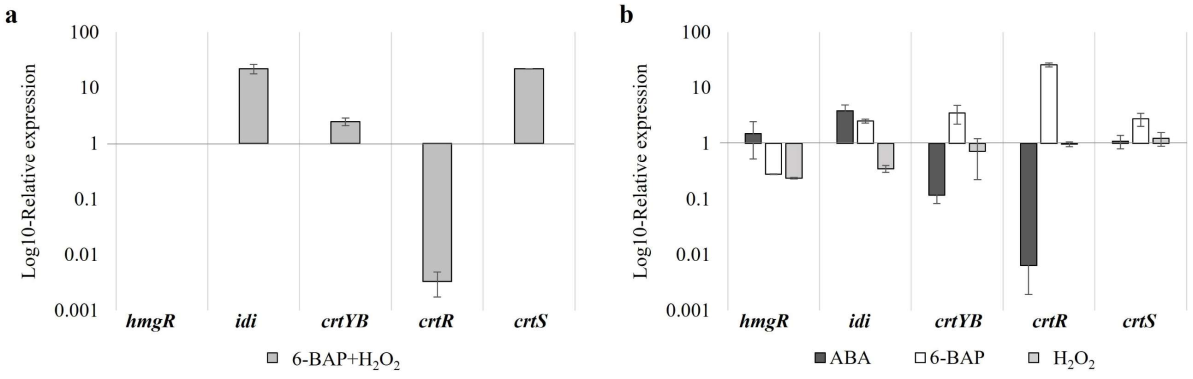

Supplemental Fig. S2. RT-qPCR expression analysis of *X. dendrorhous* ATCC 24202 genes involved in carotenoids/astaxanthin biosynthesis under stimulation conditions at 38 h fermentation. **a)** Relative expression levels of genes using 6-BAP and H_2O_2 combination treatment and **b)** relative expression levels using ABA, 6-BAP and H_2O_2 individual treatments. Each transcriptional level was normalized to the expression of the housekeeping actin gene and expressed as a function of the control treatment (relative expression = 1). The values are the mean of four independent experiments, and the error bars correspond to the standard deviations. *hmgR*, hydroxymethylglutaryl-CoA reductase; *idi*, isopentenyl pyrophosphate isomerase; *crtYB*, phytoene-β-carotene synthase; *crtR*, cytochrome P450 reductase; *crtS*, astaxanthin synthase.
